# Empirical Shaft Resistance of Driven Piles Penetrating Weak Rock

**DOI:** 10.1007/s00603-020-02228-7

**Published:** 2020-08-20

**Authors:** John W. Barrett, Luke J. Prendergast

**Affiliations:** 1 Present Address: Geosyntec Consultants, One Central Plaza, Suite 500, 835 Georgia Avenue, Chattanooga, TN 37402 USA; 2 School of Civil Engineering, University College Dublin, Newstead Building, Dublin 4, Ireland; 3 Department of Civil Engineering, Faculty of Engineering, University of Nottingham, Nottingham, NG7 2RD UK

**Keywords:** Piles, Rock, Driven, Shaft resistance

## Abstract

In this paper, an empirical relationship between the Unconfined Compressive Strength (UCS) of intact rock and the unit shaft resistance of piles penetrating rock is investigated. A growing number of civil engineering projects are utilizing steel piles driven into rock where a significant portion of the pile capacity is derived from the shaft resistance. Despite the growing number of projects utilizing the technology, little to no guidance is offered in the literature as to how the shaft resistance is to be calculated for such piles. A database has been created for driven piles that penetrate bedrock. The database consists of 42 pile load tests of which a majority are steel H-piles. The friction fatigue model is applied to seven of the pile load tests for which sufficient UCS data exists in order to develop an empirical relation. The focus of this paper is on case histories that include driven pipe piles with at least 2 m penetration into rock.

## Introduction

**Fig. 1:**
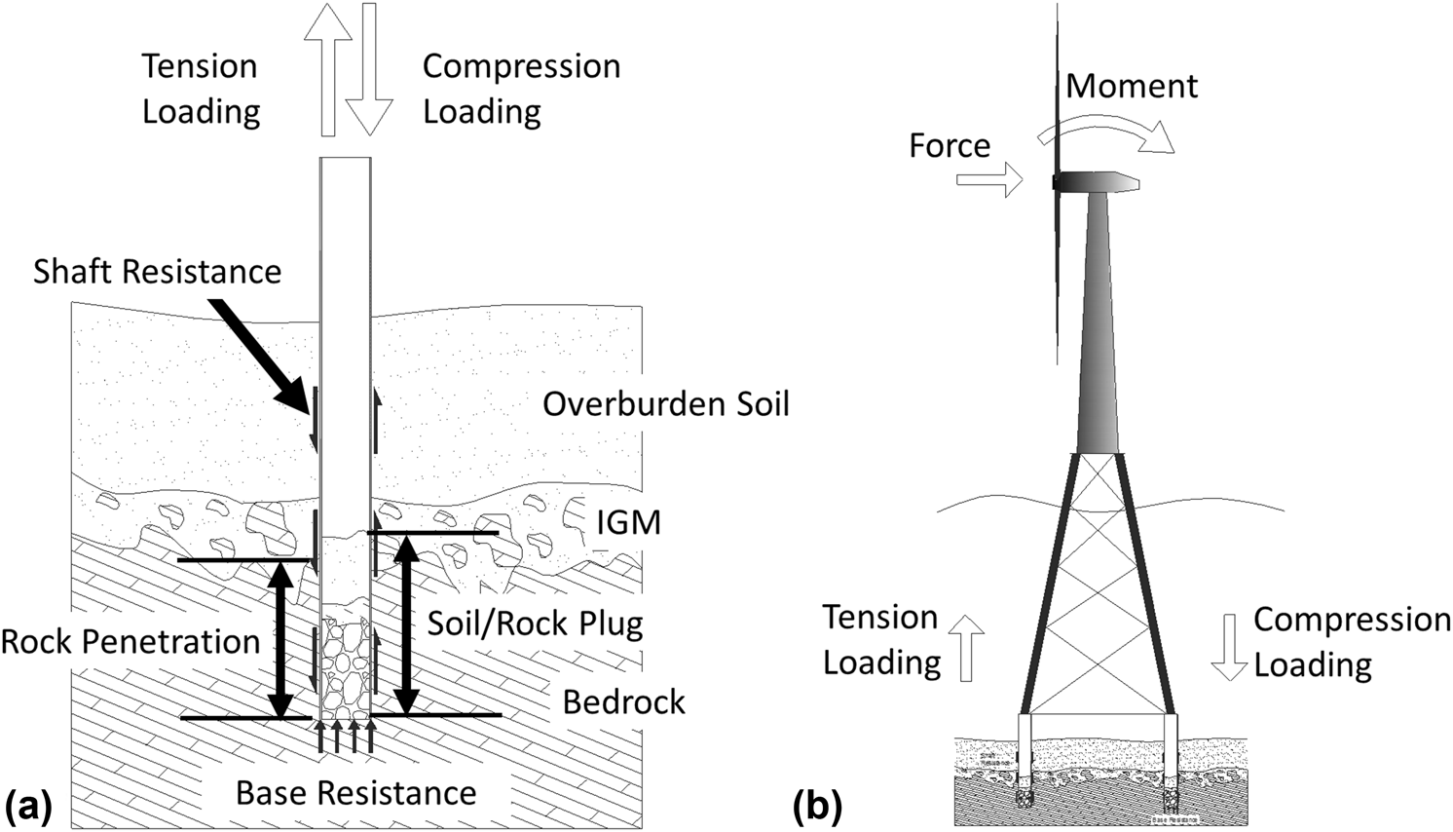
**a** Schematic of a driven pile penetrating rock; and **b** conceptual model of a wind turbine and jacket structure with piles in tension/compression

Driven piles are designed to transfer structural compressive loads through either shaft resistance, end bearing resistance, or some combination thereof, see Fig. 1a. Piles driven into rock have conventionally been treated as end bearing piles only, designed to bear on an underlying bedrock stratum. The shaft resistance due to the penetration into the weak and/or weathered rock stratum is neglected when calculating the ultimate limit state (ULS) of the pile, and the load-carrying capacity is determined based upon end bearing resistance only. For the majority of onshore projects, where the loads are primarily due to gravity, relying on the end bearing resistance of a pile bearing on rock is an acceptable approach. However, the design loading for offshore and near-shore structures require that shaft resistance be considered in order to resist uplift forces. For example, offshore wind turbines founded on jacket structures resist loading by the development of compressive resistance on one set of piles and tensile resistance on the other set as presented in Fig. 1b. This is a critical design scenario for these structures resisting significant overturning moments.

Unfortunately, very few theories or design methodologies have been proposed for estimating the shaft capacity of driven piles penetrating rock. Following the current state of practice, the engineer is left to estimate the shaft resistance in rock by utilizing static capacity methods intended for sands and clays. Due to the uncertainty in predicting the ultimate capacity, static pull-out tests are frequently required in order to confirm design assumptions during a given construction project. Even so, a significant number of case studies exists for which piles were driven to significantly penetrate the rock surface, and for many of these cases considerable pile capacity has been attributed to the shaft resistance developed within the rock.

In this paper, a model is proposed to estimate the shaft capacity of piles penetrating weak rock. The model is derived based on the results of a limited number of load tests reported in the literature, a database of which is assembled in the present work. The resulting proposed model is potentially applicable to sedimentary rock types, excluding chalk, with typical strengths of the order of 5 MPa or lower. For rocks exceeding this strength, driving is generally not possible and other design approaches such as those applicable to drilled shafts, or drilled and grouted insert piles should be adopted.

## Estimating the Unit Shaft Resistance of Driven Piles Penetrating Rock

### Traditional Approaches

A primary factor driving the decision to neglect the shaft resistance for driven piles penetrating rock is the uncertainty in the amount of damage incurred to the rock surrounding the pile during driving. Tomlinson and Woodward (2014) suggest that this is not only a result of the driven pile being analysed, but also occurs due to adjacent piles, and the damage may be so great that shaft resistance is eliminated. The process of pile penetration degrades the rock by an unknown amount and, therefore, this makes the estimation of shaft resistance around driven piles difficult (Fleming et al. 2008). According to the guideline Pile Design and Construction Practice (Tomlinson and Woodward 2014) the characteristic unit shaft resistance is determined based upon the grain size of the parent rock that has been damaged due to driving. Accordingly, coarse-grained arenaceous rocks like sandstone are thought to degrade to the consistency of a loose to medium-dense sand and the unit shaft resistance, $f_{{\text{s}}}$, is determined using methods developed for sands commonly referred to as the effective stress or $\beta$-method (Eq. 1):

1 $$f_{{\text{s}}} = K_{{\text{s}}} \sigma_{{{\text{vo}}}}^{\prime } \tan \delta_{{\text{f}}},$$

where $K_{{\text{s}}}$ is the coefficient of earth pressure, which depends on the stress history of the deposit, the volume displacement of the pile, and the pile shape/material; $\sigma_{{{\text{vo}}}}^{\prime }$ is the effective overburden stress; and $\delta_{{\text{f}}}$ is the interface friction angle between the pile and the geologic material. By observation, an implicit assumption in Eq. 1 is that $f_{{\text{s}}}$ increases indefinitely with effective overburden stress. In practice, this has been found to be false and ‘limiting skin friction values’ have been provided in guidance documents such as API RP 2A-WSD (API 2007). Following this guidance and assuming that the brittle rock has degraded to a medium-dense sand as described above, API RP 2A-WSD would limit the unit shaft resistance of the portion of the pile penetrating brittle rock to 81 kPa.

Application of Eq. 1 to driven piles penetrating rock requires some estimate of the coefficient of horizontal earth pressure, $K_{{\text{s}}}$, and the interface friction angle, $\delta$, be made. Though much research has been performed regarding the prediction of the radial stresses around piles driven through soils (e.g. Carter et al. 1986; Randolph et al. 1994), no such studies were found to guide the selection of $K_{s}$ for driven piles penetrating rock. For soils, Tomlinson and Woodward (2014) point out that the factor is influenced by: (i) the stress history of the deposit, (ii) *L*/*D* (penetration length/pile diameter), (iii) rigidity and shape of the pile, and (iv) physical properties of the pile shaft. The influence of items (ii)–(iv) is likely similar when attempting to predict $K_{s}$ for piles driven into rock. However, when driven piles penetrate rock, more factors than the stress history (or current stress state) of the rock will influence the magnitude of $K_{s}$, such as the jointing of the rock mass and the characterisation of those joints. Moreover, there is potential for arching phenomena to occur in rock. Since pile driving into rock leads to a limited zone of de-structured rock, the effective stresses acting on the pile depend on whether the de-structured material increases in volume or collapses. In the case where this material collapses, arching may occur meaning the effective stresses acting on the pile might be low. This is especially significant for increasing *h*/*D*, where *h* is the distance from the pile tip to a given soil horizon and *D* is the pile diameter (Byrne et al. 2018).

The selection of the interface friction angle is straightforward for the application of Eq. 1 to driven piles penetrating rock. As discussed above, Tomlinson and Woodward (2014) recommend that the disintegrated rock be treated as a loose to medium-dense sand and, following this guidance, the interface friction angle can be taken as that between sand and steel, which Jardine et al. (1993) have studied extensively.

Unlike coarse-grained arenaceous rocks, fine-grained argillaceous rocks such as mudstones and siltstones are thought to degrade such that they behave like clayey soils. These types of soils are normally analysed using the Total-Stress or $\alpha$-method, where the shaft resistance is calculated as in Eq. 2 (Burland et al. 2012).

2 $$f_{{\text{s}}} = \alpha c$$

2a $${\rm{For}} \quad \psi \le 1.0;\quad \alpha = 0.5 \psi^{ - 0.5}$$

2b $${\rm{For}} \quad \psi > 1.0;\quad \alpha = 0.5 \psi^{ - 0.25},$$

where $$\alpha$$ is the adhesion factor, $$c$$ is the undrained shear strength of the soil, and $$\psi$$ is the ratio of undrained strength to the effective overburden stress, $$\sigma_{{{\text{vo}}}}^{\prime }$$. Equation 2 is a straightforward application of an empirical approach where few assumptions need to be made, and has been applied with success at some sites where driven piles have penetrated rock (Thomas et al. 2011). From Eq. 2, the undrained shear strength is taken as $${\raise0.7ex\hbox{${{\text{UCS}}}$} \!\mathord{\left/ {\vphantom {{{\text{UCS}}} {2}}}\right.\kern-\nulldelimiterspace} \!\lower0.7ex\hbox{${2}$}}$$. The design UCS is traditionally derived directly from UCS tests or from correlations to point load ($$I_{{s\left( {50} \right)}}$$) tests, and $$\alpha$$ is taken as a function of $$\psi$$. An apparent flaw in this method is that while the design UCS profile is normally determined via laboratory tests on intact samples, it is reasonably understood that the true shaft resistance will be more dependent on the strength of the rock mass. In some cases, this potential problem has been addressed by modifying the design UCS profile, noting that developing design strength profiles based upon laboratory testing for which the selected samples have pre-existing but visually unidentifiable defects may produce a lower than representative strength profile (Thomas et al. 2013). For example, in the case of the piles at Port Hedland, the average UCS for the zone between 18 and 26 m below ground level (bgl) was approximately 0.55 MPa but the strength selected for design over this depth range was 1.0 MPa because visual inspection of the cores indicated higher strength (Thomas et al. 2013). Irvine et al. (2015) agree that reliance on UCS tests alone in weak rock is likely to lead to an overly conservative estimation of the strength of the rock mass. Unfortunately, no quantifiable guidance is currently available as to how much the design UCS profile should be altered according to alternative tests or methods.

While total stress approaches are normally used for argillaceous rocks, Terente et al. (2017) proposed an effective stress approach to calculate the shaft capacity of driven piles in weak rock and compared the model to a documented case study of an offshore jacket structure installed in mudstone (see Sect. 4.3). The purpose was to highlight the performance of effective stress approaches for pile design in weak rock as compared with traditional total-stress methods. The shaft capacity of three jacket piles were back-analysed from pile driving records, which revealed that the capacity was much higher than the original design predictions, which were based on an adhesion factor method. The proposed effective stress approach resulted in a closer prediction of the measured shaft capacity, albeit still with some under-prediction, which may be a result of an under-estimation of the UCS of the rock-mass as described above. Pull-out tests were not performed so there is an additional degree of uncertainty surrounding the back-analysed capacity. This study highlights the uncertainty that persists in the prediction of shaft capacity of piles in weak rocks.

Irvine et al. (2015) have suggested that piles driven into weak rock can be analysed similarly to a drilled rock-socket. However, this approach is dependent on the assumption that disturbance at the pile wall and contact between the pile and excavated rock mass is limited. Design methods for drilled-shaft rock-sockets typically estimate the unit shaft resistance to be some fraction of the UCS of the rock, as in Eq. 3 where $\alpha^{\prime}$ is a reduction factor related to the UCS and the roughness of the borehole wall, and $\varphi$ is a correction factor related to the discontinuity spacing of the rock mass.

3 $$f_{{\text{s}}} = \alpha^{\prime } \varphi \left( {{\text{UCS}}} \right)$$

For the purpose of applying these design equations to driven piles, some assumptions must be made with regard to the reduction factors $\alpha^{\prime }$ and $\varphi$. For drilled-shafts, $\alpha^{\prime }$ must give consideration to the construction methods employed, including the use of drilling fluids, the use of a roughening tool after excavation, and the pressure at which the shaft was grouted. None of these are applicable to driven piles, and the factor is purely empirical. The factor $\varphi$ has been correlated to the ratio of the elastic modulus of the rock mass to that of intact rock by Hobbs (1975). While it is likely that this ratio would influence the overall shaft resistance of a driven pile, insufficient well-documented load tests have been published for such correlations, and this factor must be taken as unity at present. In this case, Eq. 3 reduces to the same form as Eq. 2, and the unit shaft resistance is simply derived as some fraction of the UCS. Equation 3 still differs from Eq. 2 in that the factor $\alpha$ from Eq. 2 is related to the ratio of one-half of the UCS of the rock to the effective overburden stress, as opposed to $\alpha^{\prime}$ in Eq. 3, which is a constant.

Seidel and Haberfield (1995), cited in Randolph (2019), note that the unit shaft resistance of cast-in-situ piles in rock relies strongly on the interlocking mechanism between the pile and the rock surrounding it. They note that the magnitude of the unit shaft resistance will be a function of the height and roughness of rock asperities caused by drilling, which are maximum in intermediate strength rocks. An expression for calculating unit shaft resistance is proposed as shown in Eq. 4:

4 $$f_{{\text{s}}} = \psi \sqrt {\frac{{{\text{UCS}}}}{2}P_{{{\text{atm}}}} } ,$$

where *P*_atm_ is atmospheric pressure (≈100 kPa). Unlike Eq. 3, this expression suggests that unit shaft resistance increases as a function of the square root of UCS and is consistent with the data assembled by Kulhawy and Phoon (1993). The referenced study collected data of normalized unit shaft resistance against normalized shear strength for a range of geo-materials and it can be noted that the majority of data pertaining to rock fit the trend-line for *ψ* = 2, which reduces Eq. 4 to $f_{{\text{s}}} = 0.45\sqrt {{\text{UCS}}} .$

For driven piles, however, it is noted that rock minerology is expected to have a greater influence on unit shaft resistance than for the case of cast-in-situ piles (Randolph 2019). Carbonate materials tend to de-structure around piles being driven, which leads to low unit shaft resistance values. This effect varies depending on the amount of aging expected to occur, for example chalk is expected to regain shaft capacity up to a factor of five times or more (Buckley et al. 2018). In non-carbonate materials such as mudstones, higher values of unit shaft resistance can be achieved as the same degree of de-structuring is not anticipated.

### Approaches Incorporating Friction Fatigue

When driving a pile into rock, the rock must be crushed and/or disintegrated in order to accommodate the new volume of the pile being driven. Mechanical fractures are formed during driving and the rock fragments are reoriented and/or displaced as the pile penetrates the rock surface. The way in which broken rock fragments reorient themselves along the pile wall will be influenced by a plethora of variables including the rock type, diagenesis or cementation, degree of weathering, crushability of the rock, spacing and aperture of joints within the rock, and the compressive/tensile strength of the rock. Other parameters including the porosity of the material may also govern the response. It is due to this disintegration that Tomlinson and Woodward (2014), among others, have recommended the use of driven methods intended for soils be applied to driven piles penetrating rock.

The distribution of shear stress (unit shaft resistance) along driven piles has been found to be markedly different from those of drilled piles with rock-sockets. Researchers as early as Vesic (1970) observed that the unit shaft resistance along driven piles increased with depth. By contrast, Williams et al. (1980) pointed out that significant displacements were required in order to mobilize the unit shaft resistance at the bottom of drilled shafts in rock. Glos and Briggs (1983) and Williams et al. (1980) both presented load test data for drilled shafts with rock-sockets in weak rocks, and these two case histories have been compared with driven pile case studies from Matsumoto et al. (1995) and Irvine et al. (2015) in Table 1.

**Table 1:** A comparison of drilled and driven piles in weak rock

Pile type	Pile No.	Pile length (m)	Pile diameter (m)	L/D	UCS_avg_ (MPa)	Average shaft resistance (kPa)	Rock description	References
Drilled (smooth)	M9	4.2	0.66	6.4	2.3	1060	Melbourne Mudstone	Williams et al. (1980)
Drilled (smooth)	M10	7.8	0.66	11.8	3.4	470	Melbourne Mudstone	Williams et al. (1980)
Drilled (rough)	E	1.52	0.61	2.5	9.3	940	Soft Shaly Sandstone	Glos & Briggs (1983)
Drilled (rough)	W	1.52	0.61	2.5	8.4	3075	Soft Shaly Sandstone	Glos and Briggs (1983)
Driven pipe pile	T2	11	0.8	13.8	0.8	3055	Diatomaceous Mudstone	Matsumoto et al. (1995)
Driven pipe pile	1	~ 12	Unknown	Unknown	0.4–7	600	Mercia Mudstone	Irvine et al. (2015)

**Fig. 2:**
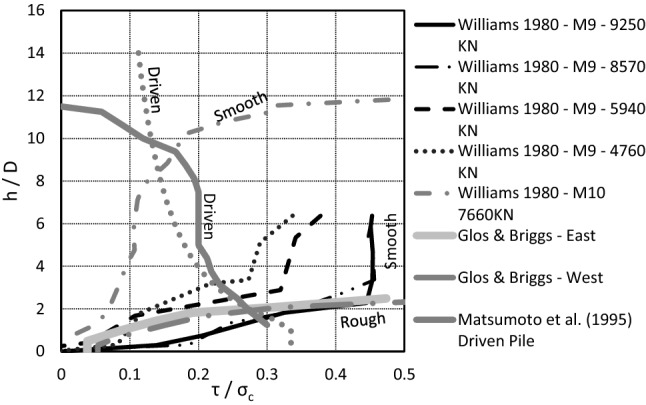
Comparison of shaft resistance for drilled shafts and driven piles in weak rocks [data modified from Williams et al. (1980), Glos III and Briggs Jr (1983), Matsumoto et al. (1995), and Irvine et al. (2015)]. ‘Driven’—an impact driven pipe pile; ‘Rough’—a drilled shaft constructed in rock for which the sidewalls have been artificially roughened through the use of a grooving tool or other measure in order to increase the shaft resistance; ‘Smooth’—a drilled shaft constructed in rock without the use of a grooving tool or other measures which can increase the shaft resistance when compared to ‘Rough’ rock sockets

Each of the case studies in Table 1 includes piles installed in weak rock. In order to draw a comparison, the shear stress distribution (*τ*) (unit shaft resistance) from the respective studies has been normalized by the UCS of the rock (*σ*_c_) and presented in Fig. 2. The *τ*/*σ*_c_ for each pile is plotted against *h*/*D*. For the tests in Williams et al. (1980), Glos III and Briggs Jr (1983), and Matsumoto et al. (1995), the shaft resistance was directly measured during the load tests using strain gauges. The data from Irvine et al. (2015) were measured using pile driving monitoring and back-analysed from the driving records. The UCS profile and pile diameter (1.27 m) for this case are obtained from Terente et al. (2017).

While the shear stress distribution or unit shaft resistance is distinctly nonlinear in all cases, and interpretation of the curves is not straight-forward, some observations can be made. For each drilled shaft shear stress distribution curve presented in Fig. 2, the highest stresses are observed at the top of the rock-sockets. This is because, in the case of smooth sockets, the concrete-to-rock adhesion is the predominant contributor to the pile’s capacity. Where displacements are sufficient to fail such bonds, abrupt decreases in the unit shaft resistance would be expected. Williams et al. (1980) present a full set of curves for pile M9 throughout loading (Fig. 2). The presentation of the curves demonstrates that mobilization of peak shear stresses or unit shaft resistance first occurs at the top of the pile. The peak shear stress is then mobilized to a depth of approximately four pile diameters at the maximum load.

In contrast with drilled piles, the unit shaft resistance for the driven piles (Matsumoto et al. 1995; Irvine et al. 2015; Terente et al. 2017) is greatest at the pile tip. The difference in the mobilization of shaft resistance for the different pile types is a function of the construction method. For driven piles, the pile–rock interface undergoes shearing as the pile is forced through rock. The pile being driven experiences peak shear stresses (mobilised shaft resistance) at the pile tip and residual shear stresses near the top of the rock. In other words, the shear strains are greater at the top of the rock socket than at the pile tip. This phenomenon is known as friction fatigue. By contrast, drilled shafts are constructed by placing concrete and/or grout within a socket excavated to full depth. When loaded, the shear stress distribution or mobilised unit shaft resistance is reliant on the stress–strain properties of the concrete and the concrete-rock interface. Because of this, it is expected that peak shear stresses would be observed near the top of the rock socket. Significant unit shaft resistance is mobilised at the deeper portions of drilled shafts only after the shaft resistance within the upper portions of the drilled shaft have been fully mobilised.

The marked differences in the shear stress distribution (unit shaft resistance) observed between driven and drilled piles should be considered when estimating pile capacity and when determining how this capacity is mobilized along the pile. For open-ended piles driven in both sand and clay, Lehane (1992) and Chow (1997) have shown that the post-installation shaft resistance along the pile can be modelled using a form of Eq. 5:

5a $$\tau_{{\text{f}}} = \sigma_{{{\text{rf}}}}^{^{\prime}} {\tan}\left( {\delta_{{\text{f}}} } \right)$$

5b $$\sigma_{{{\text{rf}}}}^{^{\prime}} = \sigma_{{{\text{hs}}}}^{^{\prime}} + {\Delta }\sigma_{{{\text{rd}}}}^{^{\prime}} ,$$

where $\sigma_{{{\text{rf}}}}^{^{\prime}}$ is the effective radial stress at failure, $\sigma_{{{\text{hs}}}}^{^{\prime}}$ is the static effective radial stress, ${\Delta }\sigma_{{{\text{rd}}}}^{^{\prime}}$ is the increase in the radial stress due to dilation, and $\delta_{{\text{f}}}$ is the interface friction angle. The post-installation shaft resistance should incorporate the degradation due to friction fatigue whereby the resistance at a given soil horizon decays with increasing pile penetration.

Several models have been proposed for this degradation. Alm and Hamre (2001) proposed an exponential relationship for the decay in shear stress (shaft resistance) along piles in both sand and clay in the context of estimating shaft resistance encountered during pile driving, which is analogous to static capacity, see Prendergast et al. (2020). Equation 6 shows the expression for estimating shear stress (shaft resistance):

6a $$\tau_{{\text{f}}} = \tau_{{{\text{res}}}} + \left( {\tau_{{\text{f max }}} - \tau_{{{\text{res}}}} } \right)e^{ - kh}$$

6b $$\tau_{{\text{f max}}} = 0.0132q_{{\text{c}}} \left( {\frac{{\sigma^{\prime }_{v0} }}{{P_{{{\text{atm}}}} }}} \right)^{0.13} \tan \delta$$

6c $$k = 0.0125\left( {\frac{{q_{{\text{c}}} }}{{P_{{{\text{atm}}}} }}} \right)^{0.5},$$

where $\tau_{{\text{f max}}}$ is the peak shear stress (shaft resistance), $\tau_{{{\text{res}}}}$ is the residual shear stress (shaft resistance), *k* is the degradation shape factor, *q*_c_ is the Cone Penetration Test (CPT) tip resistance, and *h* is the distance from the layer in question to the pile tip. *k* is specified as a function of CPT *q*_c_. Other CPT-based approaches account for the degradation due to friction fatigue by incorporating a term of the general form (*h*/*R*)^*n*^ in the expression for the effective radial stress in Eq. 5. The IC-05 approach by Jardine et al. (2005) is shown in Eq. 7 and the UWA-05 approach by Lehane et al. (2005) is shown in Eq. 8:

7 $$\tau_{{\text{f}}} = a\left[ {0.029bq_{{\text{c}}} \left( {\frac{{\sigma^{\prime }_{v0} }}{{P_{{{\text{ref}}}} }}} \right)^{0.13} \left[ {\max \left( {\frac{h}{{R^{*} }},8} \right)} \right]^{ - 0.38} + \Delta \sigma^{\prime }_{{{\text{rd}}}} } \right]\tan \delta_{{\text{f}}}$$

where *a* = 0.9 for open-ended piles in tension and 1 otherwise, *b* = 1 for compression piles, R^*^is the equivalent radius of a closed-ended pile (*R*^2^ − *R*_i_^2^)^0.5^ (*R* = external radius, *R*_i_ = internal radius), $\Delta \sigma^{\prime }_{{{\text{rd}}}}$ is the change in radial stress as a result of dilation, and *P*_ref_ = 100 kPa. This model assumes no plugging occurs during installation.

8 $$\tau_{{\text{f}}} = \frac{{f_{{\text{t}}} }}{{f_{{\text{c}}} }}\left[ {0.03q_{{\text{c}}} A_{{\text{R,eff}}}^{0.3} \left[ {\max \left( {\frac{h}{D},2} \right)} \right]^{ - 0.5} + \Delta \sigma^{\prime }_{{{\text{rd}}}} } \right]\tan \delta_{{\text{f}}} ,$$

where *f*_t_/*f*_c_ = 1 for piles in compression, and 0.75 for piles in tension. $A_{{\text{R,eff}}}^{0.3} = 1 - {\text{IFR}}\left( {\frac{{D_{i} }}{D}} \right)^{2}$ is the effective area ratio where IFR = incremental filling ratio (measure of plugging), and *D*_i_ is the internal pile diameter. The models in Eqs. 7 and 8 directly account for friction fatigue through the degradation term.

## Model Development

Twelve publications containing data for the results of a total of 44 pile load tests have been utilized for the development of a database for driven piles penetrating weak rock (Table 2). Information for each load test evaluated has been collected as comprehensively as possible. The database considers driven piles only, with rock penetration varying from approximately 2 m to greater than 18 m. At each of the sites, the UCS of the rock is determined to be low to very low. Table 2 provides the rock penetration, UCS, pile diameter, type of load test performed, measured capacity, *D*/*t* (diameter/wall thickness), and the shaft capacity as reported within each publication. It is believed that in each case the rock-sockets are below the water table; however, it is not anticipated that the degree of this submergence will significantly influence the behaviour.

**Table 2:** Summary of database of load test

Paper/Author	Rock type	No. of Piles	Rock socket length (m):	Pile diameter (m):	*D*/*t* (open ended piles)	Load test type:	Test location:	Capacity (kN)	Average shaft resistance (kPa)	Average UCS (MPa)
NGL Loading Jetty: Settgast (1980); Beake and Sutcliffe (1980)	Calcareous Siltstone/Sandstone	3	6.7 2.1 4.55	1.07 1.07 1.07	28.1 48.5 48.5	Tension Tension Tension	Offshore	4070 1765 4610	180 250 300	1.2 2.2 3.7
Noetsu bridge: Matsumoto et al. (1995)	Diatomaceous mudstone	3	8 8 8.5	0.8 0.8 0.8	66.7	Compression Compression Tension	Onshore	4700 3700 3600	184 170	0.8
Dampier Port: Beaumont and Thomas (2007)	Calcarenite/conglomerate	1	13	1.2	60.0	PDA/ CAPWAP	Nearshore	12,000 – 16,000	245 – 325	0.9
Fujairah marine jetty: Thomas et al. (2011)	Calcareous sandstone	2	18.41	0.914	48.1	Tension	Offshore	4610	87	0.9
Macleay river and floodplain: Zhang et al. (2012)	Siltstone/ mudstone interbedded with sandstone	1	3	0.75	46.9	PDA/CAPWAP	Onshore	9860	210* (> 1000)	4.3
Port Hedland: Thomas et al. (2013)	Variably cemented sedimentary rock	2	7 16.75	0.61 1.05	24.4 42.0	Tension Tension	Nearshore	5400 9500**	400 172**	1.4 1.0
Rodway and Rowe (1980)	Sandstone	3	0.5 0.75 0.92	310 UC 158 (H) 310 UC 158 (H) 310 UC 158 (H)	N/A	Tension Tension Tension	Onshore	> 750 > 750 -	– – –	Unknown
Long (1995)	Mudstone	3	Unknown	1.22	Unknown	Compression	Unknown	6300–7300	Unknown	25
Jeffers (1995)	Mercia mudstone/Keuper marl	22	Varies – interpreted from cross sections	H-Piles: 305 mm × 305 mm	N/A	Compression	Onshore	Approx. 1500	Varies	Unknown
Gannon et al. (1999)	Mudstone/sandstone	1	2	H-Pile: 305 mm × 305 mm	N/A	CAPWAP/compression	Unknown	3300 1950	– Failure not achieved	Unknown
Maertens (2002)	Basalt (weathered)	3	3.28 3.28 3.28	0.762 0.762 0.762	40.1	Compression Tension Tension	Onshore	800 – –	– – –	59

Based upon an evaluation of the unit shaft resistance of the reported case studies, an empirical design equation is proposed for calculating the effective radial stress acting on the shaft of driven piles penetrating rock in Eq. (9), termed UCD Rock Method (UCD = University College Dublin). The empirical equation incorporates friction fatigue in that the mobilized shaft resistance is reduced at a given depth below ground surface as the distance from the ground surface to the pile tip increases. The equation also builds from a relationship between the unit shaft resistance of driven piles and the UCS of the rock (or rock mass if it can be identified). It is proposed that the incorporation of this phenomenon better represents the distribution of shear stress (mobilised unit shaft resistance) along a pile that has been driven through weak rock or intermediate geo-materials. Furthermore, it is anticipated that the proposed model can be used to determine external shaft resistance only, since the mechanism of de-structuring of material within the pile likely leads to a significant difference between external and internal shaft resistance.

9 $$\sigma_{rf}^{^{\prime}} = \alpha_{0} UCS ({\raise0.7ex\hbox{$h$} \!\mathord{\left/ {\vphantom {h D}}\right.\kern-\nulldelimiterspace} \!\lower0.7ex\hbox{$D$}})^{ - \beta } \left( {\frac{1}{{1 + A_{R} }}} \right) ,$$

where $$\alpha_{0}$$ and $$\beta$$ are dimensionless scaling factors, and $$A_{{\text{R}}}$$ is the area ratio of an open-ended pile. A minimum $${\raise0.7ex\hbox{$h$} \!\mathord{\left/ {\vphantom {h D}}\right.\kern-\nulldelimiterspace} \!\lower0.7ex\hbox{$D$}}$$ value of 1 should be used in Eq. (9), as lower values would result in significantly over-predicted unit shaft resistances. For $$0 < {\raise0.7ex\hbox{$h$} \!\mathord{\left/ {\vphantom {h D}}\right.\kern-\nulldelimiterspace} \!\lower0.7ex\hbox{$D$}} < 1$$, the shaft resistance only depends on the UCS of the rock through which it is being driven, and h/D is maintained as 1 for this portion. To calibrate the dimensionless scaling factors, the model in Eq. (9) has been applied to the seven case studies in Table 3, for which the most comprehensive data are available (subset of case studies from Table 2). The $$\alpha_{0}$$ and $$\beta$$ parameters were iterated until the prediction from the model closely matched the results across seven case studies (shown by the column *Q*_s,(UCD)_/*Q*_s,(Measured)_ in Table 3 nearing unity). The interface friction value considered in the analyses was 29 degrees. For the seven case studies listed in Table 3, these factors are determined to be 0.71 and 0.45, respectively. Note that while an iterative process was implemented in this paper to obtain the dimensionless parameters for the model, it is recommended to use a more statistical-based approach. However, due to the low number of available and suitable case studies, and the likelihood of other errors related to parameter transformation (see below), it is not envisaged this process has introduced significant errors.

**Table 3:** Results of UCD rock and $\alpha$-method shaft capacity predictions

Case study	*Q*_s,(Measured)_ (kN)	Test type	*Q*_s,(UCD)_ (kN)	*Q*_s,(UCD)_/*Q*_s,(Measured)_	*Q*_s,(API predicted)_/*Q*_s,(Measured)_
Matsumoto et al. (1995)	3700	Static compressive	3616	0.98	0.64
Settgast (1980), Beake and Sutcliffe (1980)	4070	Static tensile	4426	1.10	0.53
Zhang et al. (2012)	9860	CAPWAP	10,700	1.09	0.74
Thomas et al. (2011)	4610	Static tensile	4622	1.00	1.00
Beaumont and Thomas (2007)	10,500	CAPWAP	11,600	1.12	0.52
Thomas et al. (2013)	5400	Static tensile	4621	0.87	0.38
Thomas et al. (2013)	9500	Static tensile	8510	0.90	0.73

Several key assumptions need to be highlighted when studying the relationship between the measured pile capacities, the UCS of the rock, and the interpreted shaft resistance.

1. The UCS reported may not be representative of the rock mass. All strength data are considered where available and the strength profile is a best estimate based upon this data. Unfortunately, relevant information for the determination of the strength of the rock mass is not available for most of the cases presented in Table 2. Where further descriptions are contained within the publications, these are also considered when modifying the reported UCS profile to best characterise the rock mass.
2. Where pile capacities are reported from dynamic methods, namely CAPWAP (Pile Dynamics 2014) analysis, the capacity is taken as the reported value. It should be understood that these interpreted values may be subjective. Furthermore, some discrepancy may arise in determining the portion of the pile capacity attributed to shaft resistance.
3. Insufficient information of instrumented pile load test data exists in the literature to adequately validate the theory of friction fatigue as applied to driven piles penetrating rock. For the majority of the case studies, the shear stress distribution and hence unit shaft resistance along the pile shaft is unknown.

The empirical equation developed (Eq. 9) utilized those pile load tests reported in Matsumoto et al. (1995), Settgast (1980), Beake and Sutcliffe (1980), Thomas et al. (2011), Zhang et al. (2012), Beaumont and Thomas (2007), and Thomas et al. (2013). A summary of the load tests performed and data available in each of the publications is provided in Table 3. Additionally, a comparison is made in Table 3 between the estimated pile capacity to the measured pile capacity when utilizing the UCD Rock Method (${Q}_{\mathrm{s},\mathrm{ UCD}}$) and when using the $\alpha$–Method (API 2007). The subsequent sections present case studies in order to illustrate how the developed empirical expression performs when applied to driven piles penetrating rock.

## Case Studies

In this section, an application of the proposed model to documented case studies is undertaken to demonstrate the performance of the proposed model in Eq. (9). The data from case studies 4.1 and 4.2 were used in the original model calibration and hence they are presented only to elaborate on how the model performs as compared to the existing approaches. Case study 4.3 presents the application of the model to data not considered in the model calibration and hence acts as an independent appraisal of the approach. The details and results are elaborated in the following sub-sections.

### Application to Noetsu Bridge

**Fig. 3:**
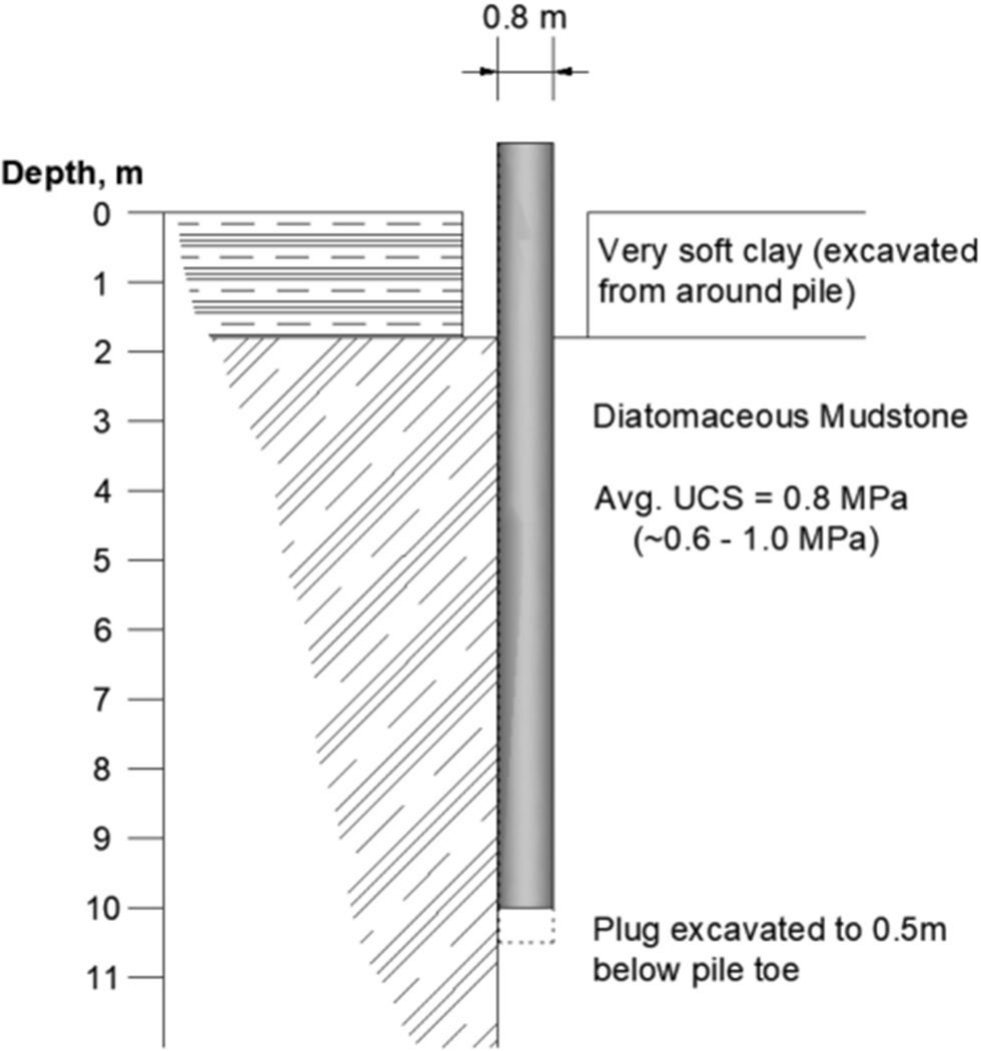
Idealized geologic profile at Noetsu Bridge (after Matsumoto et al. 1995)

Three piles were tested by Matsumoto et al. (1995) and are denoted T1, T2, and T3. T1 and T2 were compression tests with T1 completed on a pile with a plug and T2 completed with the plug drilled out to a depth of 0.5 m below the toe of the pile. T3 was the only tension test performed. The unit shaft resistance was very comparable between the three tests. The load transferred through shaft resistance for T1 was reported to be 4.2 MN. This is higher than that reported for T2 and T3, but the discrepancy arises due to the contribution of the soil plug within T1 to the measured shaft capacity. In order to quantify this contribution, a plate load test was performed using a plate of equal dimensions to the outer pile diameter. When the inner shaft capacity was assumed equal to the bearing capacity results from the plate load test, the outer shaft capacity was corrected to 3.5 MN. This is comparable to the ultimate bearing capacity of T2 (3.7 MN) and the tension capacity of T3 (3.515 MN). The idealized geologic profile for test pile T2 as developed from Matsumoto et al. (1995) is presented in Fig. 3.

**Fig. 4:**
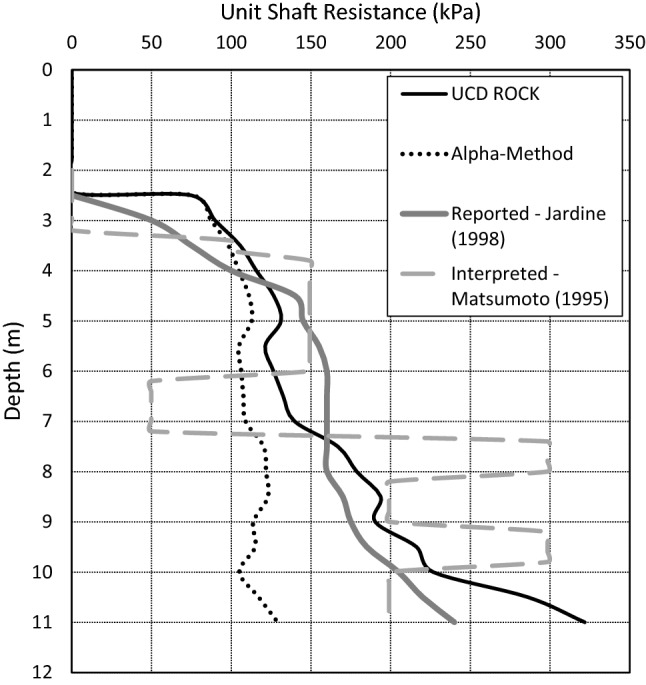
A comparison of reported unit shaft resistance and predicted unit shaft resistance for pile T2

Jardine et al. (1998) reported the shaft capacity of pile T2 to be 3.15 MN. Independent interpretation was performed herein for each of the load transfer curves presented for the Noetsu Bridge pile load tests. The interpretation of the load transfer curve for T1 is in agreement with the values as reported in Matsumoto et al. (1995) and for T2, the shaft capacity is more likely to be 3.45 MN. The interpreted shear stress distribution (unit shaft resistance) for Jardine et al. (1998) is presented in Fig. 4 as a solid grey line. A more direct interpretation from the available load-transfer curve is also presented in the figure as a dashed line. These curves are presented for the purpose of comparing the predicted unit shaft resistance. Both the prediction using the $\alpha$-method and the prediction using the empirical design equation (UCD Rock) are presented in the figure. The UCS profile at the Noetsu Bridge site is largely uniform with a range between 0.6 and 1 MPa. Similarly, the shear stress distribution (unit shaft resistance) predicted using the $\alpha$-method is largely uniform.

Using a UCS profile developed from the data presented in Matsumoto et al. (1995), the UCD Rock Method has been used to estimate the shaft capacity of the pile. Adopting an average UCS profile over the pile length that varies between a minimum of 0.6 MPa and a maximum of 0.9 MPa, the $\alpha$-method has predicted a shaft capacity of 2370 kN and the UCD Rock Method has predicted a shaft capacity of 3640 kN. When compared to the interpreted shaft capacity of 3700 kN, this equates to a predicted ratio of 0.68 and 0.98, respectively. While not surprising that the UCD Rock Method results in a close prediction (seeing as this case study was used in the model calibration), this does highlight the significant errors in estimating the shaft resistance using the α-method.

### Application to NGL Loading Jetty (Southern Arabian Gulf)

**Fig. 5:**
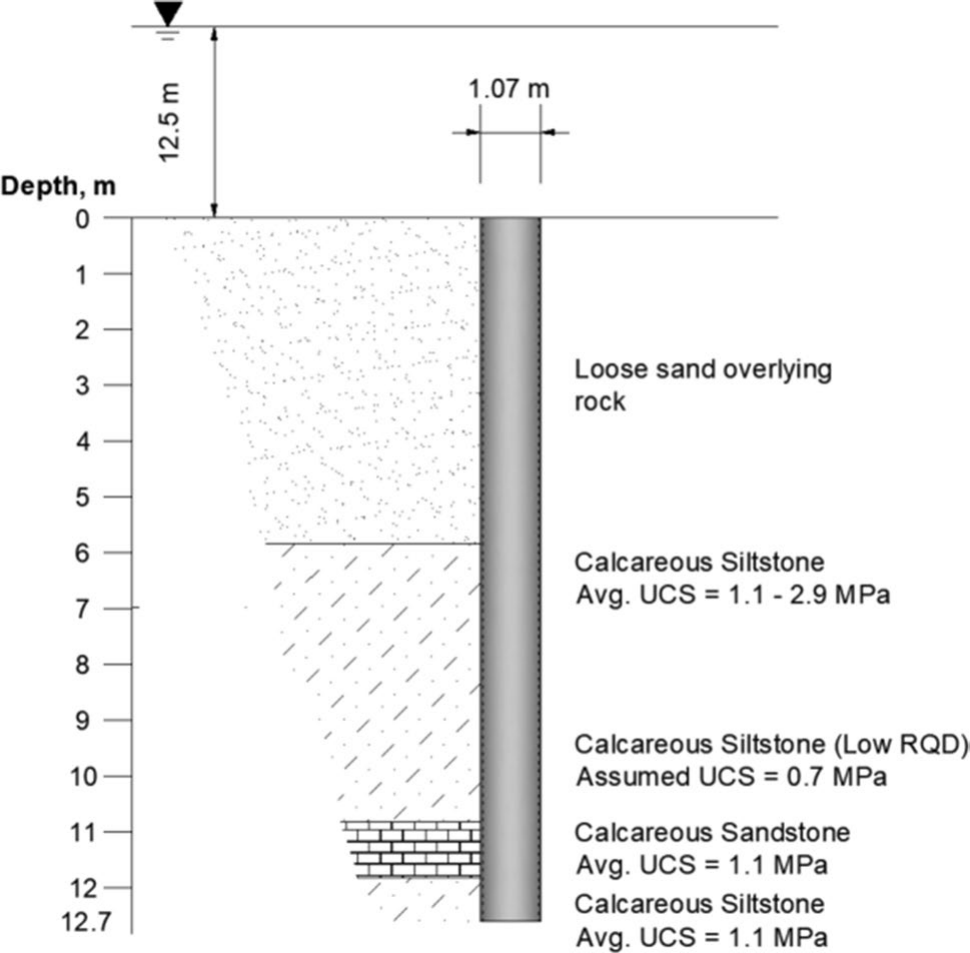
Idealized geologic profile for the NGL loading jetty (after Settgast 1980)

Three load tests were performed at the NGL loading jetty on two 1.07 m-diameter piles (Beake and Sutcliffe 1980; Settgast 1980), with *D*/*t* values of 28.1 and 48.5, respectively. The idealized geologic profile for Test Pile 1 (TP1) of the NGL loading jetty is presented in Fig. 5. Each of the test piles was driven open-ended, but TP1 was driven without the aid of predrilling while the second test pile was driven to evaluate the influence of predrilling on the driveability and shaft capacity of the pile. In this context, predrilling consists of the advancement of a pilot hole prior to driving the pile. TP1 was driven through approximately 6 m of overlying sands with little resistance, and then driven through calcareous siltstone up to a depth of 12.7 m (6.7 m into rock). Increased driving resistance was noted by Settgast (1980) at a depth of about 5 m below top of rock as the pile penetrated about 1 m of calcareous sandstone.

The data of one UCS test and four point-load tests were available for the location of TP1. Specifically, a single UCS test was available for the upper 0.1 m of the rock, and several point load tests were available to 2.1 m below the top of rock surface. The data presented by Beake and Sutcliffe (1980) and Settgast (1980) show that the average UCS of the rock across the site is between 1.75 MPa and 3.25 MPa. The upper 0.3 m of the rock had a UCS of 3 MPa, reducing to 1.75 MPa between 0.3 m and 1.2 m below the rock surface. Between 1.5 m and 2.1 m below the top of rock, the UCS reduces to 1.1 MPa. Between 2.4 m and 4.2 m depth below the top of rock, the UCS is assumed as 0.75 MPa, which is based on a single point-load test and driving resistance below 150 blows per 0.2 m penetration. At lower depths, there is no laboratory test data to enable a profile estimation, so the rock strength is based on the driving resistance, which results in an estimate of 1.1 MPa for the UCS in this area. Based upon the specific data available for the test pile location, a design rock profile has been developed with a rock strength ranging between 0.7 MPa and 3.0 MPa. Mostly, the rock strength is assumed to be near 1.1 MPa, while a reduction to 0.7 MPa is taken between 2 and 4 m below the top of rock where the RQD is reported to be 43% (otherwise, RQD > 75%).

**Fig. 6:**
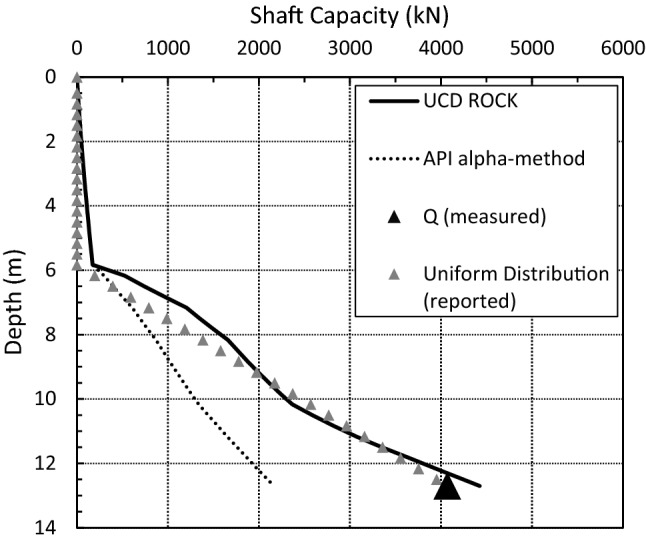
Predicted vs. observed shaft capacity at NGL loading jetty

The shaft capacity of TP1 has been estimated in Fig. 6 using the α-method and the UCD Rock Method. The actual unit shaft resistance is unknown because all strain gauges were destroyed during installation of the test piles at the NGL loading jetty. A linear distribution (termed uniform) is reported, and the measured total shaft capacity, *Q* (measured), is reported as 4070 kN (Beake and Sutcliffe 1980; Settgast 1980). The two methods, namely the α-method and UCD Rock Method, result in predicted ratios of 0.53 and 1.10, respectively. Similar to the case study in Sect. 4.1, the results are not surprising, since the results from this test were used in the calibration of the proposed model in this paper. However, the aim to highlight the relatively poor performance of the α-method at estimating the shaft capacity is achieved in the study based on the under-prediction obtained. It can, therefore, be suggested that the α-method is unsuited to estimating shaft capacity of piles driven into weak rock.

### Application to Offshore Jacket Structure

**Fig. 7:**
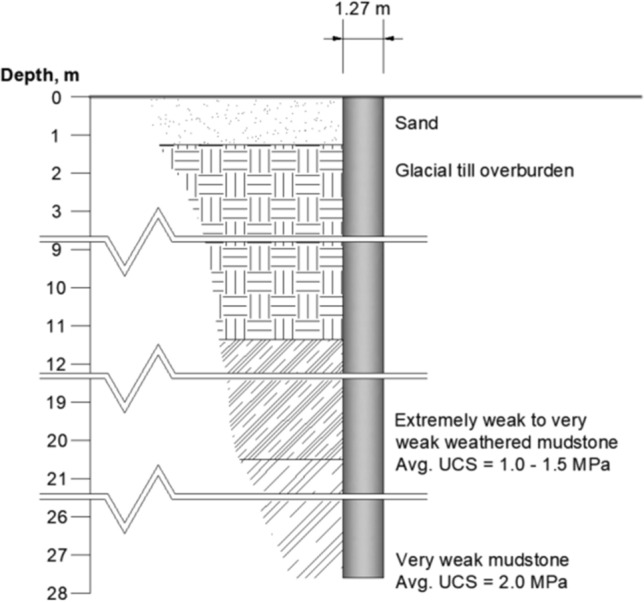
Idealized geologic profile for the case study in Terente et al. (2017)

A case study of three 1.27 m-diameter piles, with wall thicknesses of 0.045 m, installed into Mercia Mudstone is used in this section to appraise the performance of the proposed model in this paper. The case study is detailed in Terente et al. (2017) and was not used in the model calibration outlined previously. It, therefore, serves as an independent validation of the proposed model. Three piles were installed through Holocene deposits, glacial till, and Mercia Mudstone in the Irish Sea. The site profile is illustrated in Fig. 7. The top of rock is reported to be at approximately 11.3 m below seafloor (bsf). From 11.3 to 20.5 m bsf, the UCS of rock is reported to be between 1 and 1.5 MPa, and then 2.0 MPa below (Fig. 7).

**Fig. 8:**
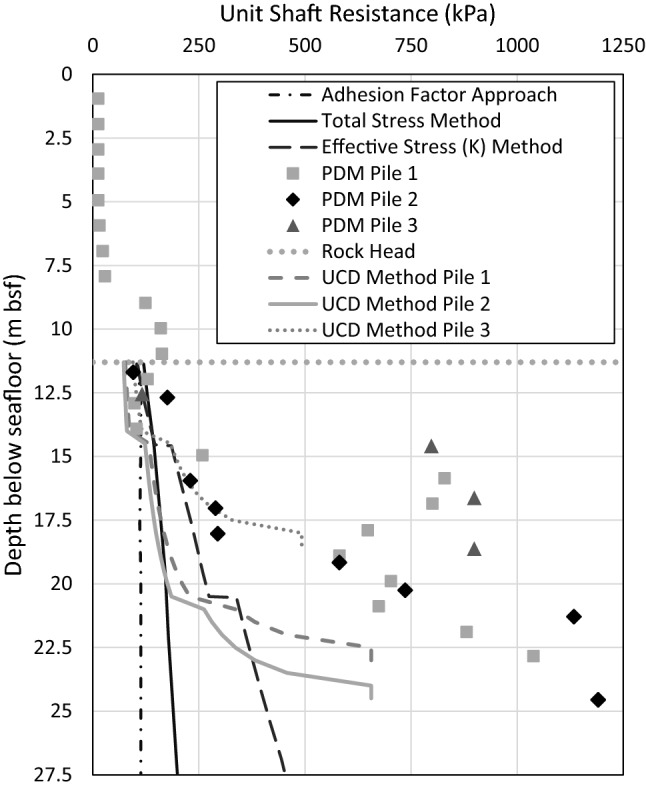
Predicted vs. observed shaft capacity of Terente et al. study

Pile installation by driving was monitored using Pile Driving Monitoring (PDM). From the reported PDM data as well as their design estimates, it appears that there is a change in the rock strength at approximately 14.5 m bsf. For that reason, a UCS of 1 MPa was assumed between 11.3 m and 14.5 m bsf, and 1.5 MPa between 14.5 m and 20.5 m bsf. The PDM measurements can be used to back-calculate the unit shaft resistance and are reported in Fig. 8.

Figure 8 presents the data from the back-figured unit shaft resistances as determined from the PDM analyses, and the predictions from the Adhesion Factor, Total Stress, and Effective Stress approaches from the study in Terente et al. (2017). Also presented are the estimations from applying the UCD Rock Method in this paper. All approaches under predict the unit shaft resistance as determined from the PDM analyses. For Piles 1 and 2, the UCD Rock Method underpredicts the unit shaft resistance determined by the Total and Effective Stress approaches to a depth between approximately 17.5 m and 20 m bsf (compared to Total Stress Approach), before deviating significantly. For Pile 3 this deviation occurs at a lower depth of approximately 15 m bsf. In spite of the underprediction in the resistance, the UCD Rock Method provides a generally better fit to the data than the methods in Terente et al. (2017), demonstrating its potential applicability to estimating soil-rock shaft resistance.

## Conclusions

The literature does not provide direct guidance on evaluating the shaft capacity of driven piles penetrating rock. Existing design methods require assumptions which tend to significantly underpredict the unit shaft resistance of such piles. Currently, the most popular design method for driven piles in rock assumes a direct relationship exists between the UCS of the rock and the unit shaft resistance along the pile. An empirical relation between the unit shaft resistance and the UCS of intact rock was derived in this paper based on the results of seven published pile load tests. The relation incorporates the phenomena observed in numerous pile load tests in dense sands and hard clays, namely friction fatigue, which indicates the shaft resistance degrades at a given depth as the distance to the pile tip increases.

The following conclusions are made from the evaluation of the developed driven piles penetrating rock database:

1. Unit shaft resistances for driven piles penetrating rock were observed to exceed the limiting maximum values reported in API RP 2A-WSD (API 2007) for the design of axial pile capacity.
2. When calibrated against and compared to seven pile load tests, reasonable predictions of the shaft capacity were made for piles driven into weak to very weak rocks. A further independent check conducted using three pile tests not used in the calibration revealed that the proposed model provides a better fit to measured data than existing models based on total stress, effective stress and adhesion factor methods.
3. The available static and dynamic load tests indicate the majority of load was mobilized through shaft resistance of the rock-socket.

The significance of these observations is that, where piles can be driven to significantly penetrate weak rocks and/or intermediate geo-materials, a large portion of the pile capacity is likely to be mobilized through the shaft resistance of the portion of the pile penetrating rock. However, the developed database of driven piles penetrating rock is insufficient to define reliable statistics. The relation between unit shaft resistance and UCS cannot be adopted as a solitary means for predicting the shaft resistance of such piles. The relation would be beneficial as a supplementary method for estimating the unit shaft resistance of the portion of driven piles penetrating rock and may be beneficial as an alternate means for evaluating a pile’s resistance to driving. Moreover, the variability in behaviour of different rock types in relation to the UCS value remains uncertain. A future study will expand the analysis conducted in this paper to a larger database of pile tests to investigate the applicability of the method to variations in rock and soil types. A statistical-based regression approach should be adopted to reliably obtain the dimensionless model parameters based on a larger data set of tests, should this become available. This is recommended as future work.

## List of symbols

*a*: Factor for open-ended piles in IC-05 approach
*A*_R_: Area ratio of open-ended pile
*A*_R,eff_: Effective area ratio of open-ended pile
*b*: Factor for compression piles in IC-05 approach
*c*: Undrained shear strength of soil (kPa)
*D*: Pile diameter (m)
*D*_*i*_: Internal pile diameter (m)
*f*_s_: Unit shaft resistance (kPa)
*f*_t_/*f*_c_: Ratio of tension to compression capacity
*h*: Distance from pile tip (m)
IFR: Incremental Filling Ratio
*k*: Degradation shape factor
*K*_s_: Coefficient of earth pressure
*P*_atm_: Atmospheric pressure (kPa)
*P*_ref_: Reference pressure (kPa)
*q*_c_: Cone Penetration Test (CPT) tip resistance (kPa)
*Q*_s,x_: Shaft capacity (kN)
*R*: Pile external radius (m)
*R*_i_: Pile internal radius (m)
*R**: Equivalent radius of closed-ended pile (m)
RQD: Rock quality designation (%)
UCS: Unconfined compressive strength (MPa)
α: Adhesion factor (total stress method)
α’: Reduction factor
α_0_: Dimensionless scaling factor UCD method
β: Dimensionless scaling factor UCD method
*δ*_f_: Interface friction angle (degrees)
Δσ’_rd_: Increase in radial stress due to dilation (kPa)
ψ: Ratio of undrained strength to consolidation stress
σ’_hs_: Static effective radial stress (kPa)
σ’_rf_: Effective radial stress at failure (kPa)
σ’_v0_: Effective overburden stress (kPa)
τ_f_: Shear stress (shaft resistance) (kPa)
τ_f,max_: Peak shear stress (shaft resistance) (kPa)
τ_res_: Residual shear stress (shaft resistance) (kPa)
